# Endoscopic vacuum therapy for postoperative esophageal leak

**DOI:** 10.1186/s12893-019-0497-5

**Published:** 2019-04-11

**Authors:** Yang Won Min, Taewan Kim, Hyuk Lee, Byung-Hoon Min, Hong Kwan Kim, Yong Soo Choi, Jun Haeng Lee, Poong-Lyul Rhee, Jae J. Kim, Jae Ill Zo, Young Mog Shim

**Affiliations:** 1 Department of Medicine, Samsung Medical Center, Sungkyunkwan University School of Medicine, Seoul, South Korea; 2 Department of Thoracic and Cardiovascular Surgery, Samsung Medical Center, Sungkyunkwan University School of Medicine, 81 Irwon-ro, Gangnam-gu, Seoul, 06351 South Korea

**Keywords:** Endoscopic vacuum-assisted closure, Esophagectomy, Leak

## Abstract

**Background:**

Anastomotic leak is the most common and serious complication following esophagectomy. Endoscopic vacuum-assisted closure (EVAC) is a promising method for treating anastomotic leak. We aimed to evaluate the efficacy of EVAC and to identify factors associated with longer treatment duration for esophageal anastomotic leak following esophagectomy for cancer.

**Methods:**

We retrospectively analyzed 20 esophageal cancer patients who had undergone EVAC for anastomotic leak after esophagectomy. The efficacy and success rates were evaluated and factors associated with longer treatment duration (≥ 21 days) were identified.

**Results:**

All 20 patients were male. Of these, 10 (50.0%) received neoadjuvant treatment and 6 (30.0%) had one or more comorbidities. The median size of fistula opening was 1.75 cm. During a median of 14.5 days of EVAC treatment, a median of 5 interventions were performed. Treatment success was achieved in 19 patients (95.0%). Neoadjuvant treatment was significantly associated with longer EVAC treatment. There was a non-significant trend toward the need for longer treatment duration for a larger fistula opening size.

**Conclusions:**

EVAC treatment is a good non-surgical option for anastomotic leak following esophagectomy. Long duration of treatment is associated with neoadjuvant treatment and a large leakage opening.

## Background

Esophagectomy has been widely used to treat several esophageal diseases including cancer. Several anastomotic complications can occur following esophagectomy. Anastomotic leak is the most common and serious complication. The leakage rates vary from 3 to 25% [1–5].

The optimal treatment for anastomotic leak remains unclear. Conservative management with nil per oral, intravenous antibiotics, and drainage and surgical and non-surgical therapies could be applied according to patient status and institutional preference [6–8]. Surgical intervention may be the treatment of choice for leaks with sepsis. In a study by Crestanello et al., the survival rate was similar between the reoperative and nonoperative groups despite the fact that the former had more severe patients [9]. If the leak is minimal and the patient is stable, conservative management can be performed to avoid a repeat surgery. When between these extremes, endoscopic treatment could be applied. Among the endoscopic treatments, the insertion of a fully covered self-expanding metal stent (SEMS) has been most extensively studied and has proven to be effective [1, 10, 11].

The essential features of esophageal leak treatment include stopping the leak and draining any collection at the anastomosis to control infection. However, coverage of the defect is occasionally difficult with SEMS implantation. In addition, SEMS treatment carries the potential risk of increasing the defect size and stent migration. Endoscopic vacuum-assisted closure (EVAC) is based on a continuous negative pressure applied to the wound with a sponge [12]. It has advantages of effective drainage of the infected fluid and acceleration of wound healing by inducing granulation tissue formation over the SEMS site [13]. EVAC was more effective for esophageal leaks than SEMS in limited cohorts [14, 15].

The present study aimed to evaluate the efficacy and safety of EVAC for esophageal anastomotic leak following esophagectomy for cancer and to identify factors associated with treatment failure and treatment duration with EVAC.

## Methods

### Patients

**Fig. 1:**
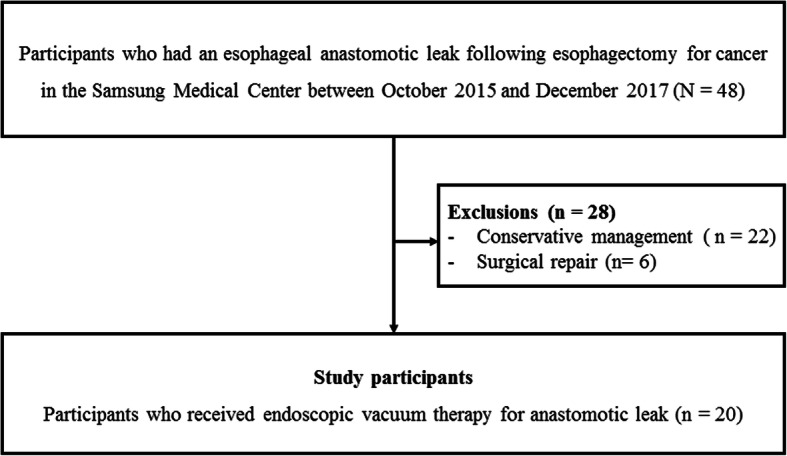
Subjects flow

This retrospective study investigated 48 patients who had an esophageal anastomotic leak following esophagectomy for cancer in Samsung Medical Center between October 2015 and December 2017. The diagnosis of postoperative anastomotic leak was made according to the associated clinical signs and symptoms and abnormal findings on endoscopy and esophagography. The associated symptoms and signs were fever, cough, neck wound swelling, and drainage of food or purulent material. Abnormal findings on endoscopy and esophagography were visualization of a wall defect and contrast leakage. About 28 patients who received only conservative management including intravenous antibiotics, nil per oral with jejunal tube feeding, and drainage (JP drain and/or chest tube) (*n* = 22) and underwent surgical repair (*n* = 6) were excluded from the study, and 20 consecutive patients who received EVAC were analyzed (Fig. 1). EVAC or surgical repair was performed among patients whose leak did not improve after conservative management and/or resulted in a septic condition. The study was approved and the need for informed consent was waived by the institutional review board of Samsung Medical Center (2017–07-068).

### Data collection and definitions

Demographic and clinical data collected from the medical records included age; sex; past medical history; comorbidities; laboratory results; surgical, endoscopic, and radiological findings; date of operation and anastomotic leak diagnosis; duration of EVAC treatment; number of EVAC interventions; use of other treatment; and mortality. Number of interventions was defined as the number of procedures including insertion, change, and removal. The Charlson comorbidity index (CCI) was used to quantify comorbidities. Anastomotic leak was diagnosed by confirmation of a mucosal defect at the anastomosis site on endoscopy. We defined treatment failure as when the anastomotic leak did not improve despite EVAC treatment and needed another treatment or the patient died due to the leak.

### EVAC treatment

**Fig. 2:**
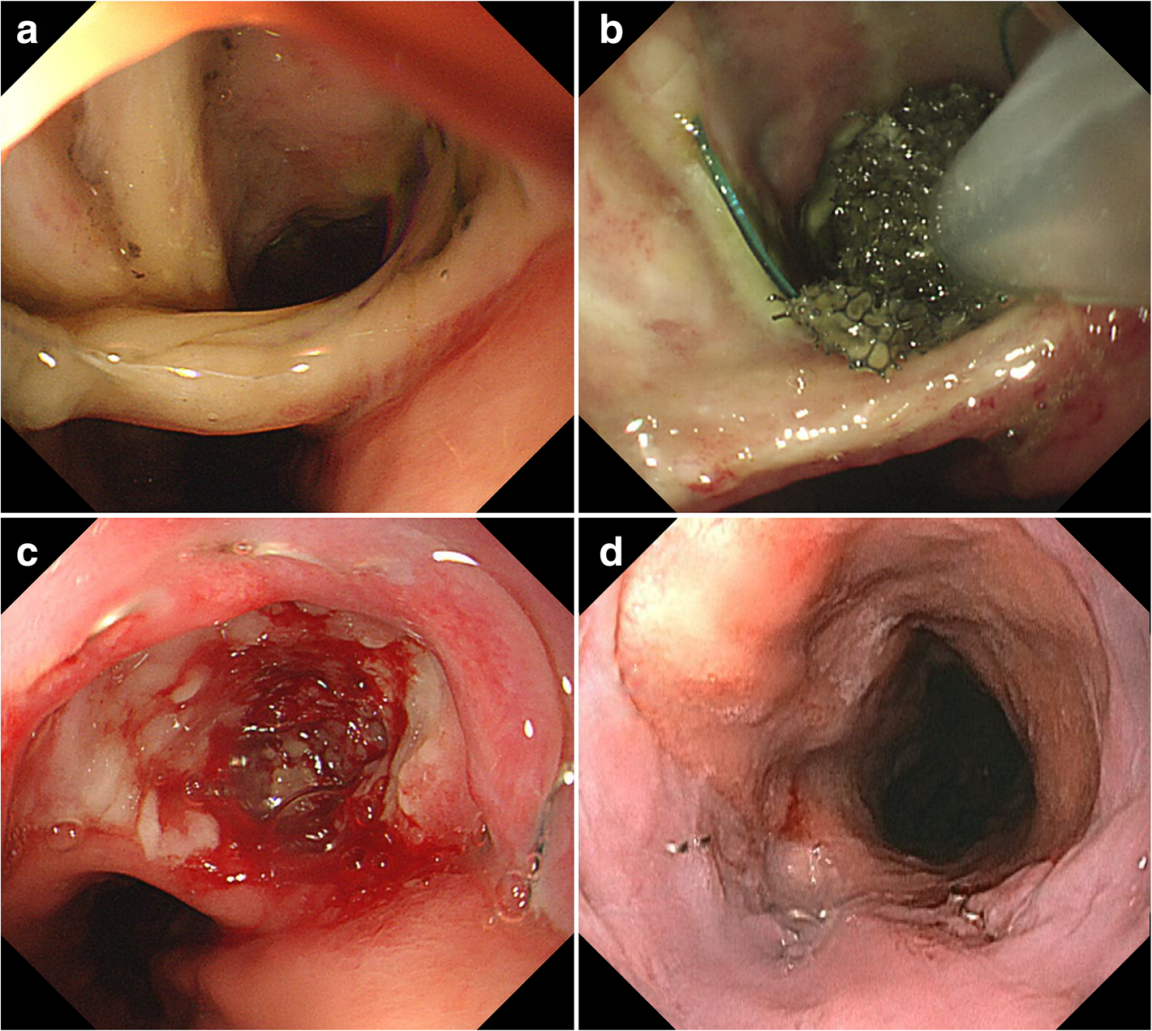
**a** A male patient was diagnosed with a postoperative anastomotic leak 7 days after Ivor-Lewis operation for esophageal cancer. The opening of the leak was estimated to be 2 cm in diameter. **b** A polyurethane sponge sutured to the tip of a nasogastric tube was inserted into the cavity of the anastomotic leak. **c** The cavity size decreased with granulation tissue and the fistula was closed after 16 days of endoscopic vacuum-assisted closure (EVAC) treatment. **d** The anastomotic leak was completely healed 31 days after treatment

The EVAC treatment began with intracavitary placement of the tip of a size-adjusted polyurethane sponge sutured to a nasogastric tube under direct vision endoscopy. A nasogastric tube is inserted via the nose and brought out through the mouth. The distal part of the tube having side holes should be removed. Then, the polyurethane sponge is sutured to the tip of the remaining tube. Trimming of the sponge is also required based on the size of the cavity. The sponge size should be smaller than the wound cavity to promote collapse and subsequent closure of the fistula. The sponge can be inserted in the wound cavity using a grasping forceps and snare. After tube placement, a continuous negative pressure of 100 mmHg which is generated by a vacuum pump was delivered to the cavity through the tube. The sponge was changed every 3–4 days according to the changes of the size and shape of the lesion. The EVAC treatment was finished when the opening was closed. Figure 2 depicts a case of a patient with successful EVAC-treated postoperative anastomotic leak.

### Statistical analysis

Categorical variables are presented as a number with percentage, and continuous variables are presented as a median with range. Nonparametric methods were used to compare the 2 groups. Continuous variables were compared using the Mann–Whitney *U* test and categorical variables were compared with the Chi-square or Fisher’s exact test. Logistic regression analysis was performed to determine the odds ratio (OR) for each variable. *P*-values < 0.05 were considered as statistically significant. Statistical analysis was performed using the SPSS version 19 (IBM Corporation, Armonk, NC, USA).

## Results

### Clinical characteristics

All enrolled patients were male, and the median age was 66.5 years (range: 53–77 years). The median white blood cell count was 8.59 × 10^3^/uL (5.59–17.48 × 10^3^/uL), and the median C-reactive protein level was 6.85 mg/dL (0.58–26.74 mg/dL). Two patients (10.0%) had respiratory failure. About 14 patients (70.0%) scored 0 on the CCI while 6 patients (30.0%) scored 1–3 points. Ten patients (50.0%) underwent neoadjuvant chemotherapy or concurrent chemoradiotherapy (CCRT). Moreover, 7 (35.0%) and 8 patients (40.0%) underwent an Ivor Lewis operation or 3-field lymph node dissection, respectively. McKeown operation was performed among 4 patients and transhiatal esophagectomy was conducted in 1 patient. Cervical and intrathoracic anastomosis were performed among 7 (35.0%) and 13 patients (65.0%), respectively. The median time to diagnosis of anastomotic leak was 12.5 days. The median size of endoscopically estimated mucosal defects was 1.75 cm. Anastomotic leak with ischemic mucosal change was noted among 16 patients (80.0%). EVAC treatment was started at a median of 3 days after the date of diagnosis. The median duration of EVAC treatment was 14.5 days, and a median of 5 interventions were performed in each patient. The median admission duration was 49 days. During the median follow-up of 213.5 days (range: 38–539 days), 7 patients (35.0%) developed anastomotic stenosis following EVAC, 19 (95.0%) were successfully treated, and 1 (5.0%) could not be treated with EVAC and died (Table 1). A 63-year-old male who received neoadjuvant CCRT and a 3-hole operation with 3-field lymph node dissection for advanced esophageal cancer was diagnosed with postoperative anastomotic leak with a 3-cm fistula opening. In spite of 6 sessions of EVAC treatment, the fistula remained patent without evidence of shrinkage. The patient and his family refused to receive more treatment, and he expired while receiving the best conservative treatment.

**Table 1:** Clinical characteristics

Variables	
Patients (*n* = 20)	
Male	20 (100.0)
Age, years	66.5 (53–77)
WBC, ×10^3^/uL	8.59 (5.59–17.48)
CRP, mg/dL	6.85 (0.58–26.74)
Organ failure	2 (10.0)
CCI score	
0	14 (70.0)
1–3	6 (30.0)
Neoadjuvant treatment	
No	10 (50.0)
Yes	10 (50.0)
Operation	
Ivor-Lewis operation	7 (35.0)
3-field lymph node dissection	8 (40.0)
McKeown operation	4 (20.0)
Transhiatal esophagectomy	1 (5.0)
Anastomosis	
Intrathoracic anastomosis	13 (65.0)
Cervical anastomosis	7 (35.0)
Anastomotic leak	
Time to diagnosis, days	12.5 (5–35)
Fistula size, cm	1.75 (0.5–3.0)
Mucosal ischemia on EGD	16 (80.0)
EVAC treatment	
Time to treatment, days	3 (0–24)
Duration of EVAC, days	14.5 (4–56)
Number of interventions	5 (2–12)
Treatment failure	1 (5.0)
Hospital stay, days	49 (19–90)
Mortality	1 (5.0)

*WBC* white blood cell, *CRP* C-reactive protein, *CCI* Charlson comorbidity index, *EGD* esophagoduodenoscopy, *EVAC* endoscopic vacuum-assisted closure

Data are shown as the median with range or number (%) of patients

### Prognostic factors associated with longer treatment

We compared the characteristics of patients with the required duration of EVAC treatment and identified the factors associated with longer treatment duration. The patients were divided into 2 groups (< 21 days and ≥ 21 days of treatment). Twelve patients (60.0%) were treated for < 21 days and 8 patients (40.0%) were treated for ≥21 days (Table 2). No significant difference was noted in the sex, age, CCI score, type of operation, level of anastomosis, time to anastomotic leak diagnosis and initial treatment, mucosal ischemia on esophagoduodenoscopy (EGD), treatment failure, hospital stay, and mortality between both groups. However, the longer EVAC treatment group had a larger number of patients who received neoadjuvant treatment (87.5% vs. 25.0%, *p* = 0.02) and a larger median size of fistula opening (2.0 cm vs. 1.0 cm, *p* = 0.05).

**Table 2:** Comparison of clinical characteristics according to the required duration of endoscopic vacuum-assisted closure (EVAC) treatment

Variables	< 21 days (*n* = 12)	≥ 21 days (*n* = 8)	*p*-value
Patients			
Male	12 (100.0)	8 (100.0)	
Age, years	67 (57–76)	63 (53–77)	0.34
CCI point			0.16
0	10 (83.3)	4 (50.0)	
1–3	2 (16.7)	4 (50.0)	
Neoadjuvant treatment			0.02
No	9 (75.0)	1 (12.5)	
Yes	3 (25.0)	7 (87.5)	
Operation			0.18
Ivor-Lewis operation	4 (33.3)	3 (37.5)	
3-field lymph node dissection	3 (25.0)	5 (62.5)	
McKeown operation	4 (33.3)	0	
Transhiatal esophagectomy	1 (8.3)	0	
Level of anastomosis			0.16
Intrathoracic anastomosis	6 (50.0)	7 (87.5)	
Cervical anastomosis	6 (50.0)	1 (12.5)	
Anastomotic leak			
Time to diagnosis, days	13 (5–35)	13 (5–24)	0.62
Fistula size, cm	1.0 (0.5–3.0)	2.0 (0.8–3.0)	0.05
Mucosal ischemia on EGD	9 (75.0)	7 (87.5)	0.62
EVAC treatment			
Time to treatment, days	3 (0–24)	3 (0–9)	0.52
Duration of EVAC, days	8 (4–18)	26 (21–56)	0.00
Interventions	3 (2–6)	8 (5–12)	< 0.01
Treatment failure	0	1 (12.5)	0.40
Hospital stay, days	45 (19–90)	59 (29–84)	0.08
Mortality	0	1 (12.5)	0.40

*CCI* Charlson comorbidity index, *EGD* esophagoduodenoscopy

Data are shown as the median with range or number (%) of patients

In univariate analysis, the OR of neoadjuvant treatment was 21.0 (95% confidence interval [CI]: 1.78–248.10, *p* = 0.02). The OR of the fistula size was 3.33 (95% CI: 0.92–12.06, *p* = 0.07), and the OR of the CCI score was 5.00 (95% CI: 0.64–39.06, *p* = 0.13) (Table 3).

**Table 3:** Univariate analysis of factors associated with a long duration of endoscopic vacuum-assisted closure (EVAC) treatment (≥21 days)

Variables	OR	95% CI	*p*-value
Age, age	0.94	0.81–1.09	0.41
CCI score			
0	1	Reference	
1–3	5.00	0.64–39.06	0.13
Neoadjuvant treatment	21.00	1.78–248.10	0.02
Operation			
Ivor-Lewis operation	1	Reference	
3-field operation	2.22	0.28–17.63	0.45
Time to diagnosis, days	1.02	0.90–1.15	0.79
Level of anastomosis			
Intrathoracic anastomosis	1	Reference	
Cervical anastomosis	7.00	0.65–75.74	0.11
Fistula size, cm	3.33	0.92–12.06	0.07
Mucosal ischemia on EGD	2.33	0.20–27.57	0.50
Time to treatment, days	0.92	0.78–1.10	0.37

*CCI* Charlson comorbidity index, *EGD* esophagoduodenoscopy

## Discussion

EVAC treatment is increasingly used for intrathoracic leakage, especially in postoperative anastomotic leaks. Several studies have reported more favorable outcomes with EVAC treatment than with SEMS placement [14, 15]. This study discusses our experience with EVAC treatment for post-esophagectomy anastomotic leaks and analyzes the predictive factors associated with failure and duration of treatment. EVAC treatment for postoperative anastomotic leak was found to be a very good treatment method with a 95.0% success rate. Nonetheless, longer EVAC treatment was required in patients who received neoadjuvant treatment. In addition, the fistula size was associated with the treatment duration.

Previous studies also showed high success rates with EVAC treatment. Brangewitz et al. reported 27 of 32 (84.4%) successful cases of EVAC treatment for esophageal defects with various causes including postoperative anastomotic leak [14]. Schorsch et al. reported 32 of 35 (91.4%) successful cases for all-cause esophageal defects and 20 of 21 (95.2%) successful cases for postesophagectomy or gastrectomy anastomotic leak [16]. Furthermore, Laukoetter et al. reported 49 of 52 (94.2%) successful cases of EVAC treatment for all-cause esophageal defects and 36 of 39 (92.3%) successful cases for post-esophagectomy or gastrectomy anastomotic leak [17]. The success rate of EVAC treatment compared to that of SEMS for postoperative anastomotic leak was significantly high in several previous studies [14, 15, 18]. In a comparative study involving 62 patients who developed an anastomotic leak following esophagectomy, EVAC showed better outcomes than stent placement and surgery [19]. Thus, EVAC is very effective for postoperative anastomotic leak, which is consistent with our results.

We identified factors predictive of longer treatment duration with EVAC. These included neoadjuvant treatment and the size of the fistula opening. Several studies reported that a large dose of radiation in neoadjuvant treatment may lead to wound healing impairment and thus, contribute to postoperative anastomotic complications [20, 21]. In this study, neoadjuvant treatment was associated with longer EVAC treatment duration. More negative pressure is required to collapse larger fistula lesions, and sufficient delivery of negative pressure is more difficult than that for smaller lesions. Therefore, longer EVAC treatment duration would be required for larger lesions.

We changed the sponge every 3–4 days according to the lesion (leak cavity) status. The drains should be changed regularly to assess the lesion status and adapt the sponge size. However, frequent changes would not be cost-effective. Thus, we modified the protocol to change the sponge every week in 2018. In a recent study showing good outcomes of EVAC, the authors also changed the sponge every 1 or 2 weeks [22]. The sponge could adhere firmly to the wound bed after prolonged placement. Therefore, weekly change seems reasonable, and a shorter interval could be considered depending on the lesion status. Future studies are needed to establish the optimal interval of EVAC change.

We experienced a 35.0% anastomotic stenosis rate after successful EVAC treatment. In all stricture cases, the patients had dysphagia and diagnoses were made through endoscopy or esophagography. All strictures were relieved with several sessions of balloon dilation. Previous studies reported lower post-interventional stricture rates than in the present study. Laukoetter et al. reported 7.7% (4 of 52 cases), Brangewitz et al. reported 9.4% (3 of 32 cases), and Schorsch reported 4.2% (1 of 24 cases) stricture rates following EVAC treatment [14, 17, 23]. However, those studies enrolled heterogeneous groups of patients with esophageal intrathoracic leaks, while we only enrolled post-esophagectomy intrathoracic anastomotic leaks. In addition, those were retrospective studies. Nevertheless, we analyzed a prospectively established database. Therefore, underestimation was possible.

The present study has some limitations. First, the sample size was small. However, it is meaningful that we reported the results for a new cohort other than the previously reported German cohorts. Moreover, we presented not only the treatment success rate but also factors associated with treatment duration. Second, it was hard to evaluate the effect of EVAC treatment because there were no comparisons with other intervention groups. Despite the lack of comparisons with other treatment modalities, the high success rate with EVAC treatment (95.0%) proves that EVAC is useful for postoperative anastomotic leak. In particular, 5% of the enrolled patients were treated with EVAC for persistent leak for which other treatments failed. Finally, this study was designed retrospectively. However, most data were extracted from prospectively collected surgical and endoscopic databases, and 2 endoscopists reviewed all photographs to ensure consistency. Therefore, bias was minimized.

The novelty of the present study relies on the validation previously observations. In addition, this is the first report to identify the factors associated with the treatment duration of EVAC.

## Conclusion

In conclusion, EVAC treatment is useful for the management of postoperative anastomotic leak following esophagectomy for cancer. Longer treatment duration is required for patients who receive neoadjuvant treatment and have large leakage openings.
